# Effect of topical application of atorvastatin gel versus hyaluronic acid on immediate loading dental implant in posterior maxilla: a randomized controlled trial

**DOI:** 10.1186/s12903-026-08081-4

**Published:** 2026-04-02

**Authors:** Amira A. M.M. Attia, Sherif M. Eissa, M. Ellayeh, H.A.M. Marzook

**Affiliations:** 1 Oral and Maxillofacial Surgery Faculty of Dentistry, Mansoura University, Mansoura, Egypt; 2 Faculty of Dentistry, Oral and Maxillofacial surgery Department, Mansoura University, Mansoura, Egypt; 3 Faculty of Dentistry, Fixed Prosthodontics, Mansoura University, Mansoura, Egypt

**Keywords:** Immediate loading, Hyaluronic acid, Atorvastatin, Bone density, Posterior maxilla

## Abstract

**Background:**

Immediate loading protocols are increasingly popular, but the complex anatomy of the posterior maxilla presents challenges. Comparative studies on the effects of hyaluronic acid (HA) and atorvastatin gel (ATV) on bone density in this region are limited. This study aimed to compare the impact of topical HA and ATV gels on bone density around immediately loaded implants in the posterior maxilla.

**Materials and methods:**

Twenty-one patients were randomly and equally assigned to three groups (*n* = 7 per group): Group I (Control): Received implants without any coating. Group II (HA group): Received implants coated with HA gel. Group III (ATV group): Received implants coated with atorvastatin (ATV) gel. Clinical evaluation of implant stability was conducted at the time of insertion and at 3 and 6 months postoperatively. Peri-implant pocket depth (PPD) and modified sulcus bleeding index (mSBI) were also assessed. Radiographic evaluation of buccal bone density (BD) was performed using cone-beam computed tomography (CBCT) immediately after implant loading and again at 6 months. All clinical and radiographic data were subjected to statistical analysis.

**Results:**

After 6 months, there was a statistically significant increase in buccal bone density in both Group II (HA) and Group III (ATV) compared to the control group (*p* = 0.001). No significant difference was found between the HA and ATV groups. There were no statistically significant differences among the three groups regarding implant stability, PPD, or mSBI (*p* > 0.05).

**Conclusion:**

HA and ATV gels increased buccal bone density but did not significantly improve implant stability or peri-implant health versus controls.

**Trial registration:**

The study was listed on www.clinicaltrials.gov with registration number (NCT06976866) on 09/05/2025.

**Supplementary Information:**

The online version contains supplementary material available at 10.1186/s12903-026-08081-4.

## Background

Dental implants are a reliable solution for replacing missing teeth, with long-term success depending on stable osseointegration. However, implant placement in the posterior maxilla remains challenging due to reduced bone density and quality, which can compromise implant stability and clinical outcomes [1]. 

To enhance bone regeneration and improve implant success in such compromised sites, biomimetic agents have been introduced to promote osteogenesis and tissue healing [2, 3]. Among these, HA and ATV are of particular interest due to their biocompatibility, anti-inflammatory, and osteoinductive properties. HA, a naturally occurring glycosaminoglycan, plays an essential role in wound healing and bone regeneration. Similarly, statins such as ATV have been shown to stimulate osteoblast differentiation and bone formation, suggesting potential applications in implantology [4–6]. 

Despite promising preclinical results, comparative clinical data evaluating the effects of HA and ATV on bone density and implant stability under immediate loading conditions are scarce. The absence of such direct comparisons represents a significant knowledge gap in current implant research [7–9]. 

Statins such as simvastatin and ATV, widely prescribed for managing hyperlipidemia, have demonstrated pleiotropic effects beyond their cardioprotective role. These include anti-inflammatory, immunomodulatory, and osteogenic actions through the stimulation of osteoblast differentiation and bone mineralization—processes essential for maintaining bone health and promoting osseointegration [10–12]. 

Similarly, HA, a naturally occurring glycosaminoglycan in connective tissues, contributes to wound healing, tissue regeneration, and bone repair due to its viscoelastic and biocompatible properties [13, 14]. Its role in maintaining periodontal and alveolar bone integrity supports its potential as a biomimetic adjunct for enhancing implant integration and soft tissue healing [15]. 

Therefore, the aim of this clinical study was to compare the effects of topically applied ATV gel versus HA gel on immediate non-functional loading (INFL) dental implants placed in the posterior maxilla. Clinical parameters included implant stability, peri-implant PPD, and mSBI, while BD was assessed radiographically using CBCT. This study directly addresses the existing knowledge gap regarding the comparative efficacy of these biomimetic agents and contributes to advancing evidence-based understanding of their role in enhancing osseointegration and bone regeneration in challenging posterior maxillary implant sites.

## Patients and methods

### Ethical statement

The study protocol was approved by the institutional review board of the Faculty of Dentistry, Mansoura University (NO. M0103023OS). The Helsinki Declaration and the guidelines set by the institutional ethics committee were adhered to in all aspects of this study’s activities. Participants in the study provided written informed consent. The study was reported in accordance with the CONSORT guidelines for clinical trials [16]. The trial was registered retrospectively on ClinicalTrials.gov (NCT06976866) on 09/05/2025 due to administrative oversight. However, the study protocol was approved by the Institutional Review Board before recruitment began, and all procedures adhered to ethical standards and the Declaration of Helsinki.

### Patient selection

Twenty-one patients were chosen for replacement of missing maxillary posterior tooth with dental implants from the out-Patient Clinic of the Oral and Maxillofacial Surgery Department at the Faculty of Dentistry, Mansoura University in the period between July 2023 and July 2024. Prior to surgery, the study’s purpose was clearly explained to all patients as well as the potential risks and complications involved.

#### Inclusion criteria

Patients with one or more missing maxillary posterior teeth with adequate alveolar ridge dimensions (minimum of 10 mm vertical bone height and at least 6 mm bone width in the edentulous posterior maxillary ridge, confirmed via CBCT), age ranging from 18 to 45 years, Patients were required to demonstrate good oral hygiene, defined as a plaque index and bleeding on probing score of less than 20%. Patient motivation and compliance were evaluated clinically prior to inclusion to ensure adherence to postoperative oral hygiene instructions. Adequate occlusion’ as confirmation of sufficient interarch space and verification of group function or anterior guidance.

#### Exclusion criteria

Included uncontrolled diabetes (HbA1c > 7%), osteoporosis, and systemic conditions impairing bone healing, pregnant individuals, individuals with any bone diseases that may impede bone healing, smokers, patients need crestal or lateral sinus lifting and individuals who exhibit parafunctional habits like bruxism and clenching.

### The patients grouping

The patients were divided randomly into three equal groups: group I (control) included 7 patients who received implants without coating them by any materials. Group II included 7 patients who received implants coated with HA. Group III included 7 patients who received implants coated with ATV gel. The patients were randomized 1:1:1 in the 3 groups and the treatments were stratified with age adjustment.

### Sample size calculation

Sample size was calculated by using G Power software (version 3.1.9.7).

In a within-between repeated measures ANOVA study, a total sample of 21 (7 per group) achieves 93.9% power to detect group time interaction with large effect size (Cohen’s f = 0.4), 5% alpha-error probability (α = 0.05), three groups, three repeated measurements, good correlation among repeated measurements (*r* = 0.5), and one non-sphericity correction (ε = 1).

#### Randomization method

For this trial with three groups [control (I), HA (II), and ATV (III)] involving 21 subjects (7 per group), a randomized block procedure [17] was used as follows:

1. A block size of 3 was chosen.
2. Possible balanced combinations with 1 C (I), 1 H (II) and 1 A (III) subjects were calculated as 6 blocks (CHA [1], CAH [2], HCA [3], HAC [4], ACH [5], and AHC [6]).
3. Blocks were chosen at random to find out the assignment of all 21 participants. The following random sequence was created: ACH [5], HCA [3], HAC [4], CHA [1], AHC, [6], CHA [1], and CAH [2]. This procedure will result in 7 participants in each of the three groups. The random allocation sequence was generated by M.H. Hend, lecturer of Public Health & Preventive Medicine, Mansoura University. The team enrolled and assigned the participants to interventions.

#### Materials

Two pieces, screw type titanium dental implant [(J dental care Dental İmplant), J Dental Care s.r.l.via Del Tirassegno, 41/N 41122, Modena, Italy] was used, HA gel ^(^Hyalubrix, Manufacturer: (fidia pharma SPA in italy) Via Ponte della Fabbrica, 3 / A 35031 Abano Terme (PD), Italy] .The concentration (10 mg/mL) and molecular weight range (1–2 MDa) of the HA gel (Hyalubrix) have been specified. and ATV gel (ATOR 20 mg, EPICO, Egypt).

#### The preparation of ATV gel

involved the precise measurement of methyl cellulose, which was then mixed with an adequate amount of biocompatible solvent (dimethyl sulfoxide). After heating the combination to a temperature of between 50 °C and 60 °C, it was shaken vigorously with a mechanical shaker until a transparent solution was achieved. Then, weighed doses of ATV (1.2 g) were added to the mixture and allowed to dissolve completely to create a homogenous mixture of medication, solvent, and polymer. This led to the creation of ATV in situ gels at a concentration of about 1.2%. ATV (1.2 g), Propylene glycol (34 g), Povidone K25 (0.67 g), Hydroxy propyl methyl cellulose (medium viscosity) (4 g), Hydroxy ethyl cellulose (N10) (3 g), and Water (57.13 g) were all present in each 100 g gel. All glassware and metals were sterilized by autoclave at 121 °C and 15 PSI for 30 min. Liquids and powders were provided as sterile material from suppliers. All preparation were conducted in a laminar flow and handled with aseptic techniques [18]. 

### Preoperative records

**Fig. 1:**
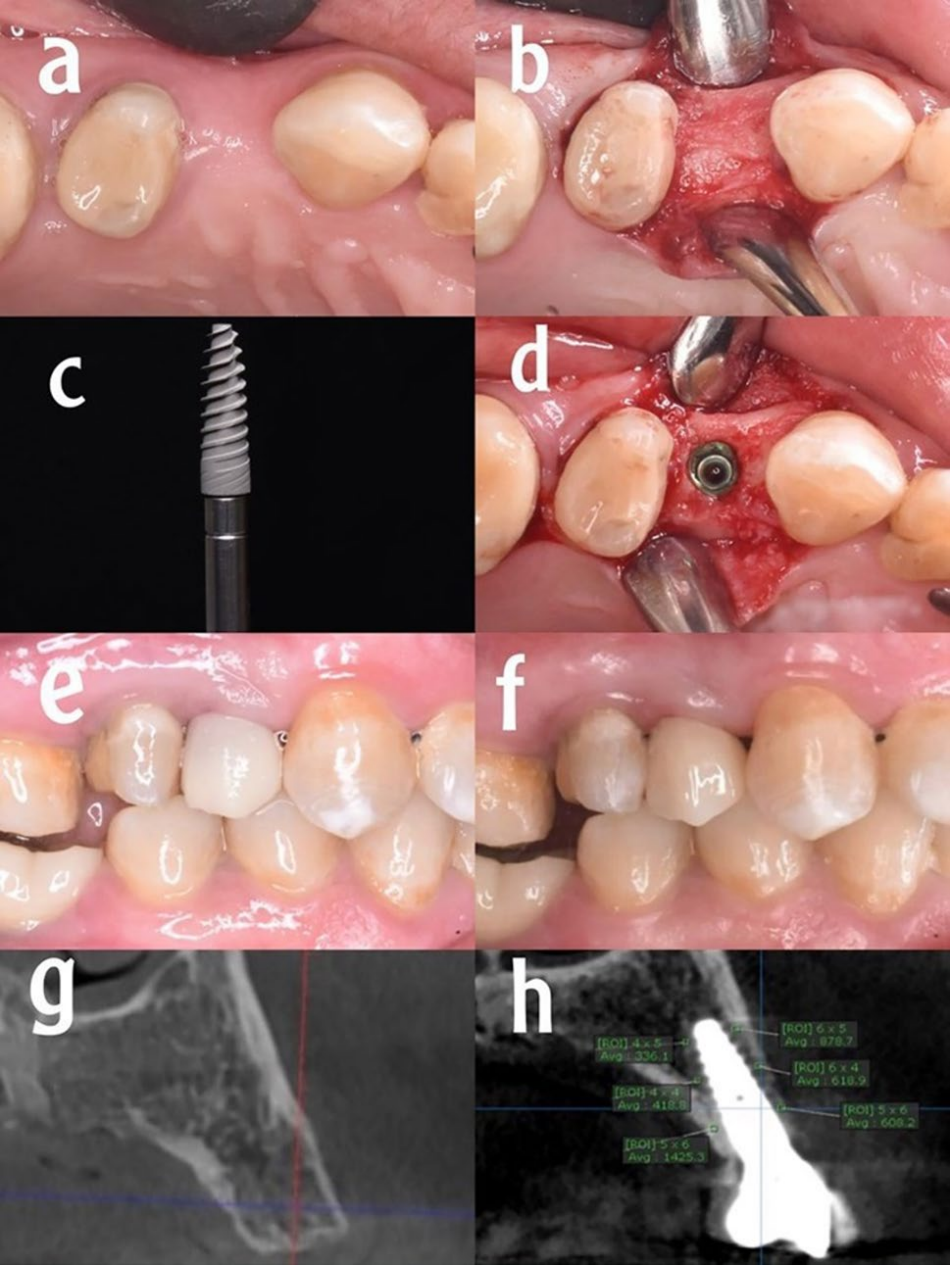
Group I (control). **a** Preoperative clinical occlusal view, (**b**) full thickness muco-periosteal flap, (**c**) the dental implant, (**d**) implant in the osteotomy site, (**e**) temporary crown, (**f**) the final restoration, (**g**) preoperative CBCT, (**h**) bone density measurements after six months postoperative

**Fig. 2:**
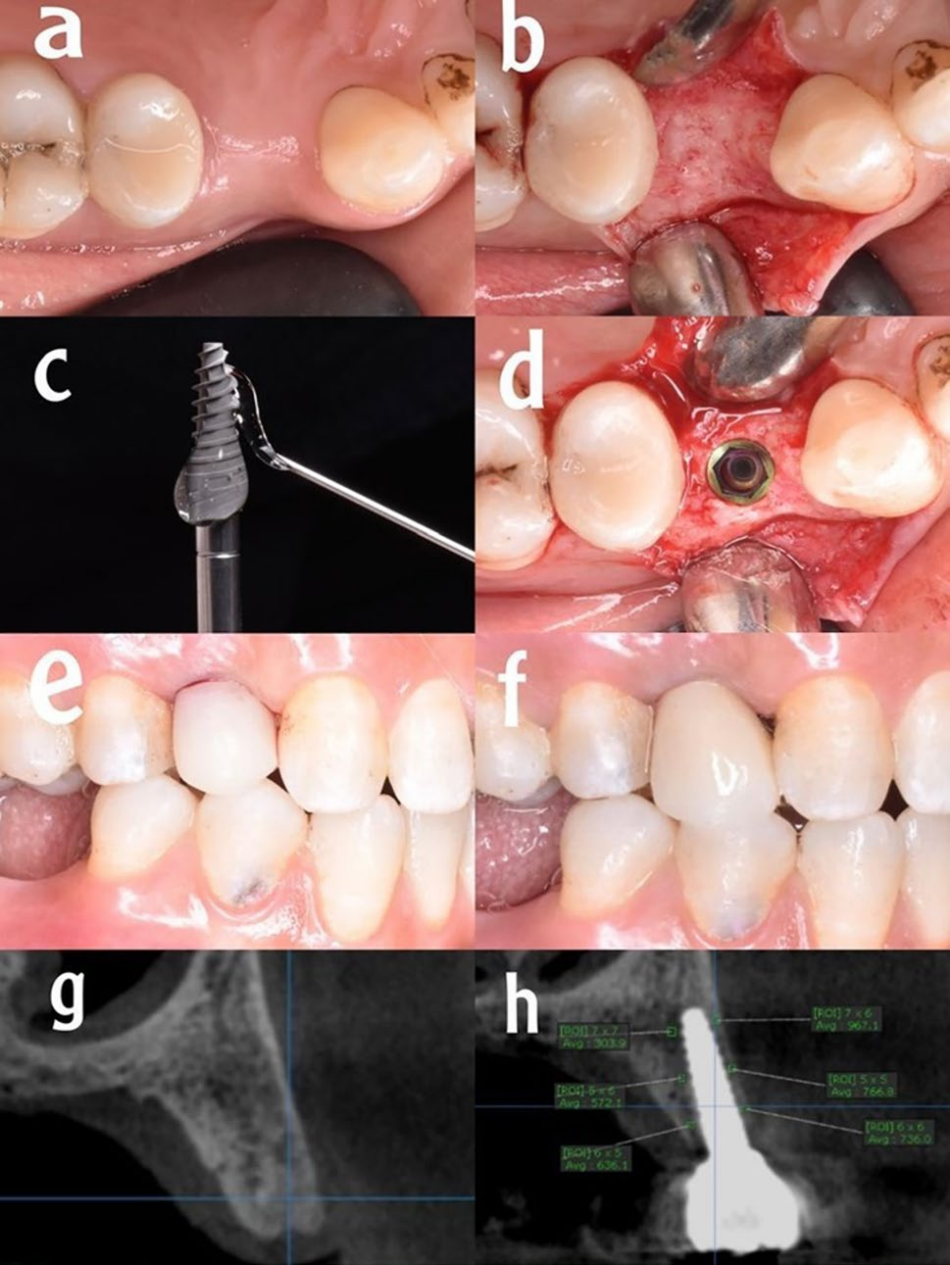
HA group. **a** Preoperative clinical occlusal view, (**b**) full thickness muco-periosteal flap, (**c**) the implant with application of HA, (**d**) the implant in the osteotomy site, (**e**) immediate temporary crown in place, (**f**) the final restoration after six months postoperative, (**g**) preoperative CBCT, (**h**) bone density evaluation after six months postoperative

**Fig. 3:**
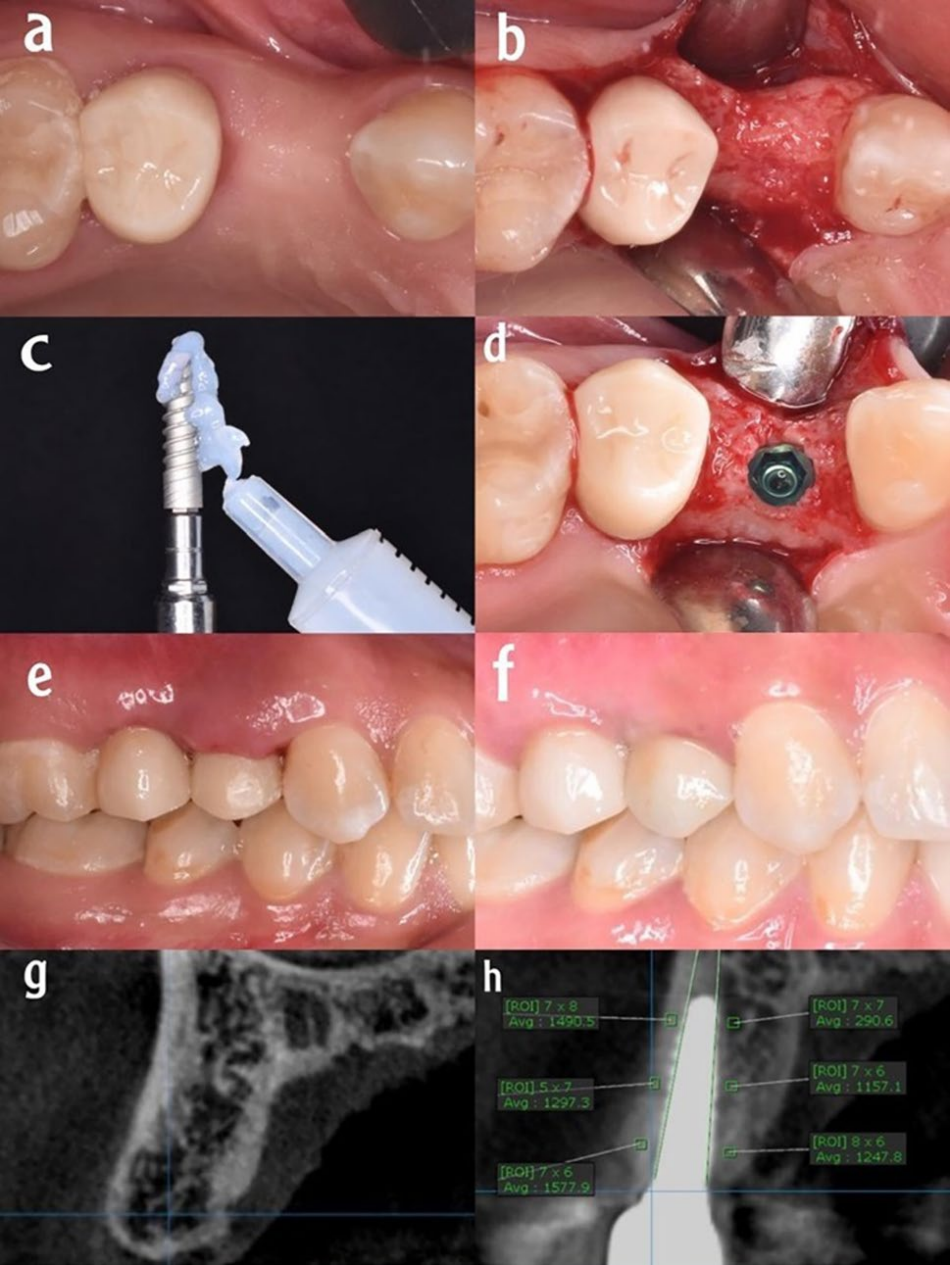
ATV group (**a**) Preoperative clinical occlusal view, (**b**) full thickness muco-periosteal flap, (**c**) the implant with application of ATV, (**d**) the implant in the osteotomy site, (**e**) temporary crown with immediate loading, (**f**) the final restoration six months postoperative, (**g**) preoperative CBCT, (**h**) bone density evaluation after six months postoperative

1. Preoperative data, including demographic information, were gathered and thoroughly documented.
2. The oral and para-oral tissues were clinically examined and palpated to guarantee proper patient selection including clinical examination and radiographic assessment (CBCT) to ensure adequate bone quality and quantity for immediate loading and to assess the planned surgical area.
3. Study casts were utilized as records for all patients to evaluate crown height space, inclination of neighboring teeth, mesiodistal width of missing tooth or teeth, and occlusion scheme.
4. Intra-oral photographs were taken preoperatively as a baseline record for all patients within the research (Figs. 1a, 2a and 3a).
5. Radiographic examination using panoramic radiograph to exclude any problems at the proposed site of implantation.
6. CBCT was taken when the patient was considered as a candidate for implant placement (Figs. 1g, 2g and 3g).

#### Surgical procedures

Strict aseptic protocols were taken prior to the start of the surgery; patients were instructed to rinse their mouths with a 0.12% Chlorhexidine (Orovex, contain Chlorhexidine Manufactured by MARCO Group Pharmaceuticals, Egypt) mouthwash for one minute. The procedures were carried out under local anesthesia (Artpharmadent, Art pharma for Pharmaceuticals Ind., Giza, Egypt) using Articaine HCL 4% with epinephrine 1:100000, administered through buccal infiltration (1.5 ml) and palatal infiltration (0.3 ml) injections. Following the administration of anesthesia, sulcular incision and elevation of a full thickness envelope muco-periosteal flap was done (Figs. 1b, 2b and 3b). Then, Preparation of the implant bed was done using an initial pilot drill to prepare the osteotomy site to the planned depth, with ample irrigation of normal sterile saline to achieve the appropriate angulation and inclination based on preoperative evaluations, followed by a series of sequential drills from smallest to largest sizes until reaching the final drill.

Procedures were done for group I without coating materials (Fig. 1c), for group II implants were coated with HA (Fig. 2c), and for group III implants were coated with ATV (Fig. 3c). The coating process was standardized as follows: a consistent amount of the respective gel was applied evenly to the implant surface immediately prior to insertion to ensure uniform coverage. The gel was applied to the implant surface and the osteotomy site immediately before the final insertion. To minimize the wash-out effect, irrigation was strictly limited during the final seating of the implant.All coatings were applied by the same experienced operator to reduce variability. The implants were then inserted directly into the prepared osteotomy sites without any waiting period.A ratchet wrench was employed to complete the implant’s final placement one millimeter below the crest as advised by the manufacturer (Figs. 1d, 2d and 3d). All included implants achieved a primary stability of at least 35 N.cm to ensure adequate primary stability. Subsequently, healing abutments were attached to the implants. Then interrupted sutures were used to reposition the flap after placement of the healing abutments using 4/0 polypropylene suture (4/0 Polypropylene Monofilament, G.M.S, Alexandria, Egypt) to maintain clean healthy soft tissue around the implant. Following the completion of suturing, the healing abutment was removed, and subsequently, an open tray impression technique was used for all patients. Once the impression procedure was finished, the healing abutment was reinserted until the temporary restoration was made.

### Postoperative care

All patients were directed to keep good oral hygiene and have a liquid or semi-liquid diet while avoiding any solid textured food for 2 weeks postsurgically then gradually return to normal diet [19]. postoperative antibiotic clindamycin (Clindam each capsule contains: 300 mg clindamycin, Manufactured by SIGMA, Egypt) 300 mg was prescribed twice daily for 5 days. For analgesic we used paracetamol (Panadol each tablet contains: 500 mg paracetamol, Manufactured by Alexandria pharmaceuticals and chemical industries, Egypt) 500 mg with a maximum of 4000 mg in any 24 h period. After two to three days of implant placement, the healing abutment was removed, and a milled polymethyl methacrylate (PMMA) screw-retained temporary restoration was fabricated and delivered to all patients for 6 months (Figs. 1e, 2e and 3e). Then after 6 months, the final screw-retained zirconia was placed (Figs. 1f, 2f and 3f).

### Clinical evaluation

Implant stability was evaluated at the time of implant insertion and at 3 and 6 months postoperatively using the Osstell^®^ device (Osstell, Integration Diagnostics, Savadaled, Sweden). Resonance frequency analysis (RFA) was conducted by attaching a smart peg transducer to the implant, and the frequency response analyzer measured the implant stability quotient (ISQ) values. ISQ measurements were taken from four directions—buccal, lingual/palatal, mesial, and distal—and the average ISQ value was recorded for each implant to improve measurement reliability [20]. Also, probing pocket depth (PPD) using a graduated periodontal probe with gentle pressure to prevent excessive tissue damage and intrusion into surrounding healthy tissue. The probe was inserted at four sites around the implant (buccal, palatal, mesial, and distal) in alignment with the implant’s vertical axis until the probe’s blunt edge reached the base of the sulcus and measurements were rounded to the nearest 0.5 mm, and the average of the four measurements was recorded [21], and mSBI was done at four specified locations around the implant (bucally, palatally, mesially, distally) to assess the presence of peri-implant mucosal inflammation, and scores from 0 to 3 which indicates (no bleeding when probing adjacent to the implant, isolated bleeding with visible blood spots, confluent red line of blood along the gingival margin, heavy or profuse bleeding, respectively) [22], were evaluated after three and six months postoperatively.

### Radiographic evaluation

CBCT scans were taken pre-operatively, immediate post-operatively, and 6 months after implant loading for measuring and evaluation of the relative bone density around the dental implant. All patient scans were performed using a Planmeca ProMax^®^ 3D unit (Planmeca OY, Helsinki, Finland). with a fixed imaging parameter at each scan. Then, OnDemand3D software (Version 1, CyberMed, Seoul, South Korea) was used to analyze all DICOM data.

### Bone density recording

The greyscale bone measuring tool was utilized to gather all density measurements from a bucco-palatal view of the cross-sectional plane [23]. All CBCT data were analyzed using OnDemand3D software (Version 1, CyberMed, Seoul, South Korea). The greyscale bone density measurements were obtained with the software’s built-in bone measuring tool on cross-sectional bucco-palatal views, at standardized locations 1 mm from the implant surface. Six measurements per implant (three on the buccal and three on the palatal side) were averaged to assess changes over time. Three readings were taken on the buccal side (coronal, middle, and apical third) of the implant fixture, followed by three similar readings on the palatal side (Figs. 1h, 2h and 3h). All measurements were conducted by the same examiner. The average greyscale values [intra-examiner reliability for greyscale measurements was high (Cronbach’s α = 0.92)] of the three readings on the same side were computed. Subsequently, bone densities from the immediate postoperative and 6-months postoperative tomographs were compared. Standardization of bone density measurements was ensured using the implant itself as a constant reference. Measurements were taken at three fixed anatomical levels: the cervical, middle, and apical thirds of the implant, using the software’s orientation tools to replicate the exact cross-sections at all follow-up intervals.

### Statistical analysis

Data analysis was conducted using SPSS software, version 26 (SPSS Inc., PASW statistics for Windows version 26, Chicago: SPSS Inc.). Qualitative data was represented using numbers and percentages. For non-normally distributed data, quantitative data was described using the median (minimum and maximum), while for normally distributed data, the mean ± standard deviation was utilized after confirming normality through the Shapiro-Wilk test. The significance of the results was determined at the level of ≤ 0.05. Chi-Square, Fisher exact test, and Monte Carlo tests were employed to compare qualitative data between groups when applicable. The Kruskal-Wallis test was used for comparing more than 2 studied groups with non-normally distributed data. Wilcoxon signed rank test was utilized for comparing two time points. One-way ANOVA test was conducted for comparing more than 2 independent groups, followed by the post hoc Tukey test to identify pairwise comparisons. Repeated measures ANOVA test was employed to compare more than two time points, with the post hoc Tukey test used for pairwise comparison.

## Results

**Fig. 4:**
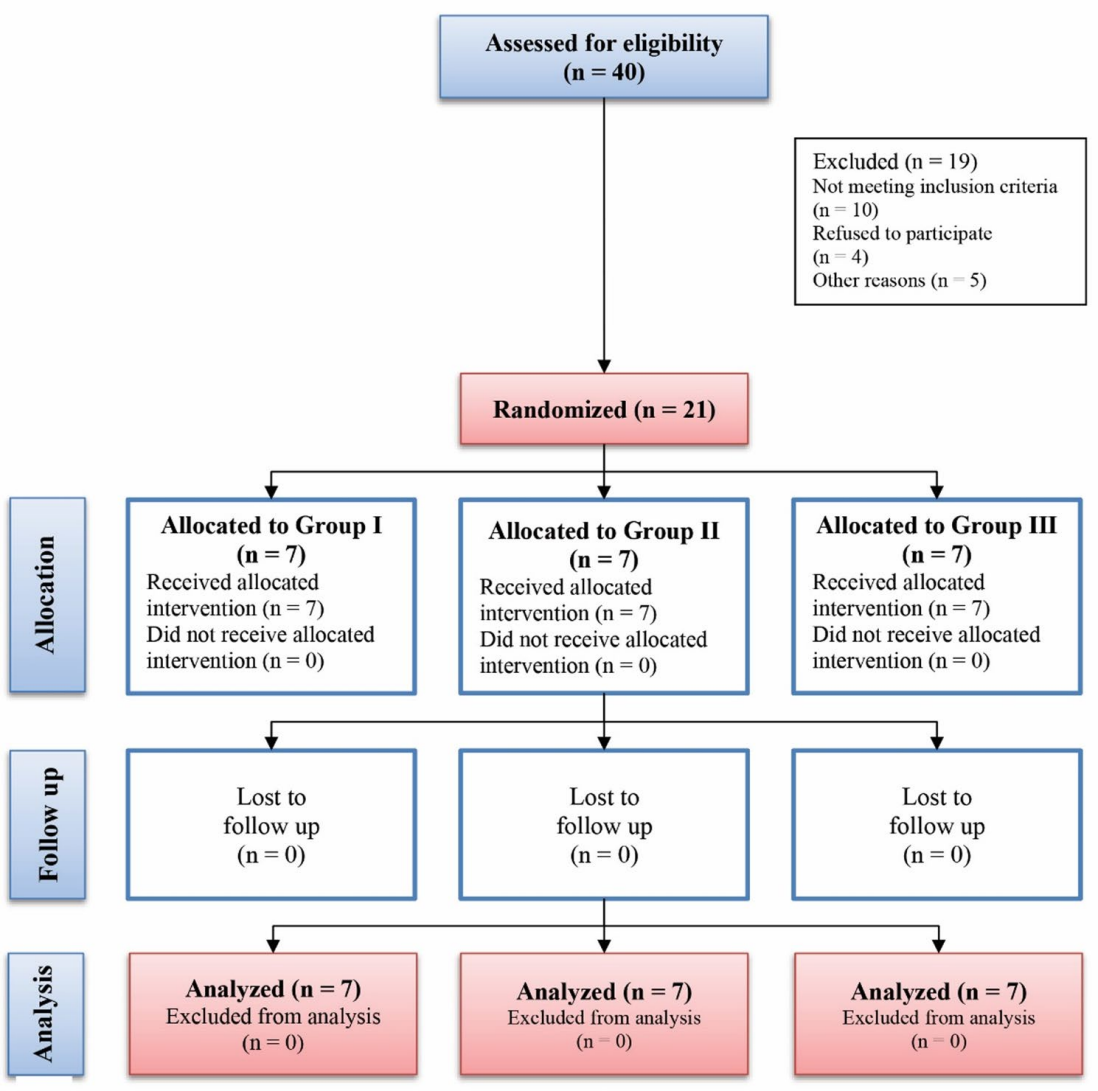
Flow Chart according to the CONSORT guidelines

Out of the 40 patients assessed for eligibility, 19 did not meet inclusion criteria and were excluded and 21 subjects were randomized into three study groups This study included twenty-one patients who received twenty-one implants in the posterior maxilla. See the CONSORT Flow Diagram depicted in (Fig. 4) for further details. No statistically significant difference was found among the three groups regarding age and sex (*P* = 0.791 and *p* = 0.558, respectively) and implant specifications (length, width) (*p* = 0.907 and *p* = 0.848 respectively). We only put one implant in the first molar area in each group and the rest of the implants in the premolar area. All inserted implants recorded a survival rate of 100% without any signs of peri-implantitis or failure as shown in (Table 1).

**Table 1:** Comparison of demographic data, implant length and width in the three studied groups

	Control Group(I)	HA Group (II)	ATV Group (III)	Test of significance	Within group significance
	*N* = 7	*N* = 7	*N* = 7		
Age / years (mean ± SD)	33.29 ± 5.88	32.86 ± 6.67	35.14 ± 7.19	F = 0.237 Pg = 0.791	P1 = 0.905 P2 = 0.605 P3 = 0.526
Sex Male Female	3(42.9) 4(57.1)	2(28.6) 5(71.4)	4(57.1) 3(42.9)	MC = 1.17 Pg = 0.558	P1 = 0.613 P2 = 0.613 P3 = 0.317
Implant Length	13.64 ± 1.38	13.64 ± 1.38	13.92 ± 1.43	F = 0.098 Pg = 0.907	P1 = 1.0 P2 = 0.706 P3 = 0.706
Implant diameter	3.95 ± 0.32	4.04 ± 0.32	4.04 ± 0.32	F = 0.167 Pg = 0.848	P1 = 0.623 P2 = 0.623 P3 = 1.0

Data distributed as ( mean ± SD)

SD: standard distributed p: probability

One Way ANOVA test (F) for comparing between the three studied groups according to (age / years) and implant (length / diameter)

Monte Carlo test (MC) for comparing between the three studied groups according to Sex (male/ female)

*Statistically significant if *p* ≤ 0.05

Pg: significance between the three groups

p1: difference between groups I and II, p2: difference between groups I and III

p3: difference between groups II and III

### Clinical results

#### (I) Implant stability

As shown in Table 2, there were no statistically significant differences in implant stability between the three groups at any time point (immediate, 3 months, or 6 months postoperatively). However, all groups showed a statistically significant increase in mean stability over time (*p* = 0.001).

**Table 2:** Comparison of implant stability between the three studied groups immediately postoperative and along evaluation intervals

Group Time	Control Group (I)	HA Group (II)	ATV Group (III)	Test of significance (One Way ANOVA test) #	Within group significance
	*N* = 7	*N* = 7	*N* = 7		
Immediate postoperative	70.42 ± 3.87	70.86 ± 4.14	70.14 ± 3.23	F = 0.064 Pg = 0.938	P1 = 0.834 P2 = 0.889 P3 = 0.727
After 3 months postoperative	74.14 ± 4.09	75.86 ± 4.06	75.0 ± 2.94	F = 0.368 Pg = 0.697	P1 = 0.402 P2 = 0.673 P3 = 0.673
After 6 months postoperative	76.42 ± 4.04	77.57 ± 4.35	77.86 ± 3.72	F = 0.245 Pg = 0.786	P1 = 0.603 P2 = 0.517 P3 = 0.896
Repeated Measures ANOVA test ##	F = 122.54 Pt = 0.001*	F = 78.93 Pt = 0.001*	F = 93.17 Pt = 0.001*		
	P_a_=0.001* P_b_=0.001* P_c_=0.001*	P_a_=0.001* P_b_=0.001* P_c_=0.001*	P_a_=0.001* P_b_=0.001* P_c_=0.001*		

Data distributed as (mean ± SD)

Pt: difference between time intervals

Pg: difference between the three studied groups

# One Way ANOVA test (F) for comparing between the three studied groups

## Repeated Measures ANOVA test for comparing time intervals (immediate postoperative ,3 and 6 months postoperatively

*statistically significant if *p* ≤ 0.05

p1: difference between groups I and II, p2 : difference between groups I and III

p3: difference between groups II and III

p_a_: difference between pre-operative and immediate post-operative readings

p_b_: difference between pre-operative and after 6 months post-operative readings

p_c_: difference between immediate post-operative and 6 months post-operative readings

#### (II) PPD

As shown in Table 3, no statistically significant differences in PPD were observed between the three groups at either 3 or 6 months postoperatively. However, each group demonstrated a statistically significant increase in mean PPD over time (*p* ≤ 0.001), with all groups showing higher values at 6 months compared to 3 months, indicating a time-related change in soft tissue response around the implants.

**Table 3:** Comparison of PPD between the three groups studied and during follow up periods

Group Time	Control Group (I)	HA Group (II)	ATV Group (III)	Test of significance (Kruskal Wallis test)	Within group significance
	*N* = 7	*N* = 7	*N* = 7		
3 months post-operative	1.43 ± 0.44	1.36 ± 0.38	1.50 ± 0.41	kw = 0.209 Pg = 0.813	P1 = 0.750 P2 = 0.750 P3 = 0.526
6 months post-operative	2.21 ± 0.57	2.29 ± 0.39	2.36 ± 0.38	kw = 0.173 Pg = 0.842	P1 = 0.772 P2 = 0.564 P3 = 0.772
Mann Whitney U test	Z = 7.78 pt = 0.001*	Z = 13.0 pt < 0.001*	Z = 9.29 pt < 0.001*		

Data expressed as (mean ± SD)

Kruskal Wallis test (kw) for comparing between the three studied groups

Mann Whitney U test (Z) for comparing between 3 months and 6 months post-operative

*Statistically significant if *p* ≤ 0.05

Pt: difference between time intervals

Pg: difference between the three studied groups

p1: difference between groups I and II, p2: difference between groups I and III

p3: difference between groups II and III

#### (III) mSBI

As shown in Table 4, there were no statistically significant differences in mSBI between the three groups at either 3 or 6 months postoperatively. However, each group demonstrated a statistically significant decrease in mSBI from 3 to 6 months (*p* < 0.05), suggesting improved peri-implant soft tissue health over time.

**Table 4:** Comparison of mSBI between the three studied groups and during follow up period

Group Time	Control Group (I)	HA Group (II)	ATV Group (III)	Test of significance (Kruskal Wallis test)	Within group significance
	*N* = 7	*N* = 7	*N* = 7		
3 months postoperative Median (min-max)	2(1–2)	2(1–2)	2(1–2)	KW = 0.949 Pg = 0.406	P1 = 0.248 P2 = 0.248 P3 = 1.0
6 months postoperative Median (min-max)	1.0(1.0–1.0)	1.0(1.0–1.0)	1.0(1.0–2.0)	KW = 0.955 Pg = 0.404	P1 = 0.656 P2 = 0.492 P3 = 0.378
Wilcoxon signed rank test	Z = 2.41 Pt = 0.016*	Z = 2.38 Pt = 0.017*	Z = 2.38 Pt = 0.017*		

Data distributed as median (min-max)

Kruskal Wallis test (KW) for comparing different studied groups I, II and III

Wilcoxon signed rank test (Z) for comparing between 3 months and 6 months postoperative

Pt: difference between time intervals

Pg: difference between the three studied groups

p1: difference between groups I and II p2: difference between groups I and III

p3: difference between groups II and II

### Radiographic evaluation

As shown in Table 5, there were no statistically significant differences in palatal bone density among the three groups at any time point (preoperative, immediate postoperative, or 6 months; *p* > 0.05). However, each group demonstrated a statistically significant increase in bone density over time (*p* = 0.001), with values rising consistently from baseline through to 6 months postoperatively.

**Table 5:** Assessment of BD values for palatal surfaces between three studied groups and during follow up periods

Group Time	Control Group	HA Group	ATV Group	Test of significance (One Way ANOVA) #	Within group significance
	*N* = 7	*N* = 7	*N* = 7		
Preoperative	320.28 ± 89.09	323.14 ± 52.84	296.29 ± 36.94	F = 0.375 Pg = 0.692	P1 = 0.937 P2 = 0.489 P3 = 0.441
Immediate postoperative	427.0 ± 85.69	465.42 ± 77.35	478.28 ± 76.97	F = 0.045 Pg = 0.956	P1 = 0.880 P2 = 0.885 P3 = 0.767
After 6 months	558.0 ± 97.82	572.14 ± 52.59	548.0 ± 86.18	F = 0.180 Pg = 0.837	P1 = 0.748 P2 = 0.556 P3 = 0.788
Repeated Measures ANOVA test ##	F = 38.74 Pt = 0.001*	F = 61.71 Pt = 0.001*	F = 74.1 Pt = 0.001*		
	P_a_=0.001* P_b_=0.001* P_c_=0.001*	P_a_=0.001* P_b_=0.001* P_c_=0.001*	P_a_=0.001* P_b_=0.001* P_c_=0.001*		

Data expressed as (mean ± SD)

P: probability

# One Way ANOVA test (F) for comparing the three studied groups

## Repeated Measures ANOVA test for comparing the time intervals (immediate, 3 months and 6 months postoperatively

*Statistically significant if *p* ≤ 0.05

Pt: difference between time intervals

Pg: difference between the three studied groups

p1: difference between groups I and II, p2: difference between groups I and III

p3: difference between groups II and III

p_a_: difference between pre-operative and immediate post-operative readings

p_b_: difference between pre-operative and after 6 months post-operative readings

p_c_: difference between immediate post-operative and 6 months post-operative readings

As shown in Table 6, there were no statistically significant differences in buccal bone density between the three groups at the preoperative and immediate postoperative stages (*p* > 0.05). However, at the 6-month assessment, groups II and III showed significantly higher bone density compared to group I (*p* = 0.001), while no significant difference was observed between groups II and III.

Within-group comparisons revealed a statistically significant increase in buccal bone density at each time point across all groups (*p* = 0.001).

**Table 6:** Assessment of BD values for buccal surfaces between three studied groups and during follow up periods

Group Time	Control Group	HA Group	ATV Group	Test of significance (One Way ANOVA) #	Within group significance
	*N* = 7	*N* = 7	*N* = 7		
Preoperative	330.14 ± 77.09	349.14 ± 44.21	293.57 ± 35.52	F = 1.82 Pg = 0.189	P1 = 0.528 P2 = 0.232 P3 = 0.08
Immediate postoperative	572.0 ± 61.67	570.29 ± 53.68	575.57 ± 58.07	F = 0.015 Pg = 0.985	P1 = 0.956 P2 = 0.909 P3 = 0.866
After 6 months	670.57 ± 35.89	802 ± 81.1	813.42 ± 70.55	F = 10.31 Pg = 0.001*	P1 = 0.001* P2 = 0.001* P3 = 0.748
Repeated Measures ANOVA test ##	F = 168.92 Pt = 0.001*	F = 137.07 Pt = 0.001*	F = 478.74 Pt = 0.001*		
	P_a_=0.001* P_b_=0.001* P_c_=0.001*	P_a_=0.001* P_b_=0.001* P_c_=0.001*	P_a_=0.001* P_b_=0.001* P_c_=0.001*		

Data expressed as (mean ± SD)

P: probability

# One Way ANOVA test (F) for comparing the three studied groups

## Repeated Measures ANOVA test for comparing the time intervals (immediate, 3 months and 6 months postoperatively

*Statistically significant if *p* ≤ 0.05

Pt: difference between time intervals

Pg: difference between the three studied groups

p1: difference between groups I and II, p2: difference between groups I and III

p3: difference between groups II and III

p_a_: difference between pre-operative and immediate post-operative readings

p_b_: difference between pre-operative and after 6 months post-operative readings

p_c_: difference between immediate post-operative and 6 months post-operative readings

## Discussion

The present study aimed to compare the effects of topically applied HA and ATV gels on bone density and implant stability in immediately loaded posterior maxillary implants. The results confirmed the study hypothesis, demonstrating that both HA and ATV enhanced buccal bone density compared to controls, although no significant differences were observed in implant stability or peri-implant soft tissue parameters.

Immediate loading protocols have gained prominence in implant dentistry due to their ability to reduce treatment time and surgical interventions while meeting patient demands for faster prosthetic rehabilitation [24]. 

In the present study, all implants were immediately loaded with non-functional provisional restorations kept out of occlusion by 1 mm. This approach aligns with the findings of Singh et al. [25], who reported that INFL reduced occlusal stress during the healing phase and improved peri-implant bone density compared to immediate functional loading (IFL).

The present investigation focused on the use of two bioactive gels—ATV and HA—applied topically to the implant surface to evaluate their effect on clinical and radiographic outcomes following INFL in the posterior maxilla. While previous literature has suggested that surface bioactivation with agents like HA and statins may enhance osseointegration [8, 12], our study specifically examined their effects under INFL conditions.

All implants achieved 100% survival, with no clinical signs of failure. Primary stability is essential for successful osseointegration [26, 27], and our results showed no statistically significant difference in ISQ values among the three groups at baseline, 3 months, and 6 months. This indicates that neither HA nor ATV significantly influenced implant stability over time, although a slight increase in mean ISQ values was observed in the HA and ATV groups compared to the control group, consistent with Wessam et al. [28].

Moreover, the increase in ISQ values over time across all groups suggests progressive bone remodeling and maturation around the implants—a finding supported by Vollmer et al. [29], who reported peak osseointegration at six months post-placement.

PPD increased slightly within each group from 3 to 6 months but remained within the clinically acceptable range (max. 3 mm). The changes were not statistically significant between groups. This mild increase may be attributed to postoperative soft tissue remodeling, surgical trauma, or flap reflection during implant placement [21, 30]. These findings are in agreement with Singh et al. [31], who also reported similar peri-implant tissue responses over time.

The marked increase in buccal bone density observed immediately after placement is likely attributed to mechanical bone compaction during insertion and beam hardening artifacts inherent in CBCT imaging of metallic implants, rather than immediate biological bone formation.

mSBI scores decreased significantly from 3 to 6 months in all groups, suggesting effective plaque control and improved peri-implant tissue health. This outcome reflects patients’ adherence to oral hygiene instructions and supports Kim et al. [32], who emphasized the role of oral hygiene in maintaining soft tissue health around implants.

Radiographically, BD measurements revealed no significant differences among the three groups preoperatively or immediately postoperatively. However, after six months, the buccal bone density was significantly higher in the HA and ATV groups compared to the control. No differences were observed in the palatal aspect. These findings suggest a localized effect of the gels on buccal bone remodeling.

The enhancement in buccal BD in the HA group aligns with Elhadidi et al. [8], who demonstrated improved buccal bone density following topical HA application. Similarly, Yazan et al. [33], reported HA’s ability to preserve osteoinductive growth factors, thereby promoting bone regeneration. Conversely, Boot et al. [34], found no significant effect of HA on peri-implant bone density, highlighting inconsistencies in the literature potentially due to differences in HA formulation, concentration, or study design.

In the ATV group, our findings correspond with ElDemerdash et al. [9] who reported increased osteoid and trabecular bone formation with statin application. Other studies by Moraschini et al. [35] and Kellesarian et al. also support the osteogenic effect of statins, showing enhanced osseointegration and bone contact. However, some reports, such as by Xianqi et al. [36], suggest statins may promote osteoclastogenesis and bone resorption under certain conditions, indicating a dose-dependent or context-specific.

The clarification of immediate change is primarily attributed to mechanical and radiographic factors rather than biological ones. The presence of the metallic implant within the bone creates a ‘compacting effect’ on the surrounding trabeculae during insertion, and more significantly, it produces ‘beam hardening artifacts’ in the CBCT image, which can artificially increase the measured radiopacity (gray values) in the peri-implant area and the immediate baseline was used to calibrate subsequent measurements (3 and 6 months), and that the true biological effect of HA and Atorvastatin is reflected in the progressive density changes observed in the follow-up periods compared to this baseline.

Interestingly, despite no significant differences between HA and ATV groups in the 6 months, both showed significant improvements in buccal bone density compared to the control. Even the control group showed increased BD over time, likely due to the INFL protocol, as supported by Singh et al. [25].

The increased density in the buccal wall is clinically significant as it may contribute to long-term stability of the labial bone plate, potentially preventing ridge resorption and maintaining optimal soft tissue esthetics.

We also acknowledge that our bone density measurements were based on grayscale values derived from CBCT imaging. These values are device- and protocol-dependent and cannot be directly equated with true bone mineral density. Thus, while CBCT provides valuable relative data, its limitations in absolute quantification should be considered.

### Study limitations

First, the small sample size of seven patients per group and the relatively short follow-up period of six months limit the generalizability of our findings. While the results indicate a potential benefit of HA and ATV gels on bone density, further studies with larger sample sizes and extended follow-up durations are necessary to confirm these findings and assess the long-term effects of these treatments on implant stability and peri-implant health.

Second, the absence of histological analysis limits direct confirmation of osseointegration. Additionally, clinicians performing stability assessments were not blinded to the gel treatment groups, which may introduce measurement bias. Finally, although we assessed intra-examiner reliability for the greyscale measurements, yielding a high Cronbach’s alpha of 0.92, we did not perform additional validation analyses such as factor analysis or test-retest reliability. Future studies should consider these validation metrics to strengthen the reliability and applicability of the measurement tool.

## Conclusion

In this randomized clinical trial, topical application of hyaluronic acid and atorvastatin gels significantly increased buccal bone density around immediately loaded implants in the posterior maxilla compared with uncoated implants. No significant differences were observed between the groups regarding implant stability or peri-implant soft tissue parameters over six months, indicating that the observed benefits were primarily radiographic. These findings suggest that both agents may serve as adjunctive biomaterials to enhance bone quality in low-density maxillary sites.

## Supplementary Information

Supplementary Material 1.

## Data Availability

The data sets used and/or analyzed during the current study are available from the corresponding author on reasonable request.

## Abbreviations

HA: Hyaluronic acid
ATV: Atorvastatin
PPD: Pocket depth
mSBI: Modified sulcus bleeding index
BD: Bone density
ISQ: Implant stability quotient
INFL: Immediate non-functional loading
IFL: Immediate functional loading
