# Supplementary material for: Effect of topical application of atorvastatin gel versus hyaluronic acid on immediate loading dental implant in posterior maxilla: a randomized controlled trial
Source: BMC Oral Health. 2026 Apr 2;26:638. doi: 10.1186/s12903-026-08081-4 (PMC13063705; doi:10.1186/s12903-026-08081-4)
Supplement: Supplementary file 1 — Supplementary Material 1. [file 12903_2026_8081_MOESM1_ESM.docx]

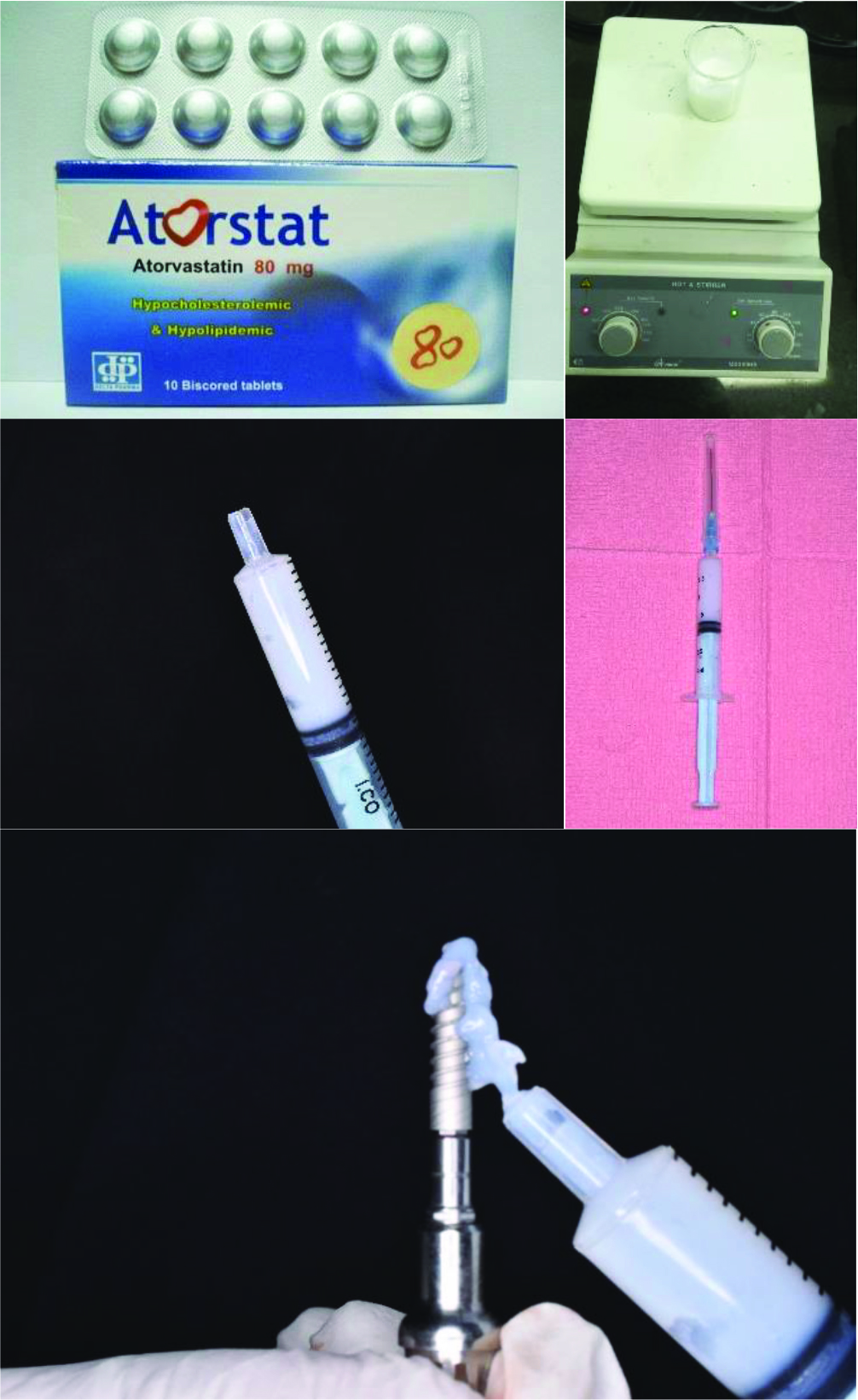


**Fig (5)** showing atorvastatin gel preparation and application over the implant


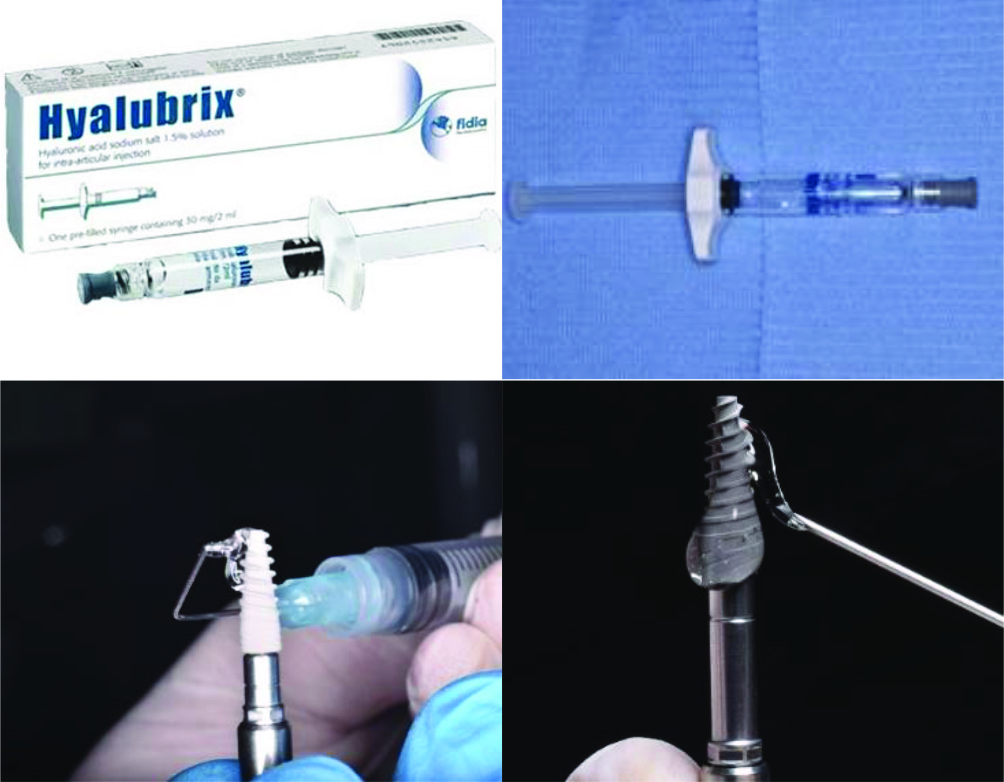


**Fig (6)** showing hyaluronic acid gel and application over the implant
